# Hemorrhagic myocardial infarction: detection using susceptibility weighted phase imaging

**DOI:** 10.1186/1532-429X-14-S1-P30

**Published:** 2012-02-01

**Authors:** James W Goldfarb, Usama Hasan, Wenguo Zhao, Jing Han

**Affiliations:** 1 Research and Education, St Francis Hospital, Roslyn, NY, USA; 2 Biomedical Engineering, SUNY Stony Brook, Stony Brook, NY, USA; 3 Medical School, New York College of Ostopathic Medicine, Old Westbury, NY, USA

## Summary

In this study we determined the normal range of high-pass filtered image phase values in a group of normal subjects for all sixteen segments of the heart. The range and standard deviations varied significantly across the heart, with less variance in septal segments. In a group of acute myocardial infarction patients, phase values in hemorrhagic myocardial infarction were significantly less than normal segments and well depicted as hypointense lesions in filtered-phase images.

## Background

Myocardial hemorrhage in patients with acute myocardial infarction (AMI) is a form of severe reperfusion injury. Detection has been shown with T2-weighted (T2W) and T2*-weighted magnitude images and is closely associated with microvascular obstruction (MVO). Limitations of the T2W technique include the prerequisite of myocardial edema as well as adequate visualization of myocardial edema to detect a hypointense infarct core. Overall quality of T2*-weighted images across all segments of the myocardium limits its usage for evaluation of the entire left ventricle. Susceptibility-weighted phase images have been shown to be sensitive for the detection of hemosiderin in microbleeds of the brain and should be an effective means of detecting myocardial hemorrhage.

## Methods

15 healthy control subjects and 11 AMI patients were studied at 1.5 T. The comprehensive study protocol included cine, T2-weighted, T2*-weighted, resting perfusion and late gadolinium-enhanced imaging. High-pass filtered phase images were reconstructed from breath-hold multi-echo T2*-weighted acquisitions (Echo delay time(TE) =2.4-15.5ms, 2.2ms spacing) over the entire left ventricle. ROIs were drawn for each subject in each of the 16 AHA myocardial segments and the mean phase and standard deviation were recorded. Normal variations in myocardial phase across echo delay time and anatomical segment were compared in the control group using ANOVA and normal ranges as mean+-2*standard deviations were calculated. Analysis of the phase measured with the same control group protocol across echo times in AMI patients was plotted and compared to normal ranges.

## Results

**Figure 1:**
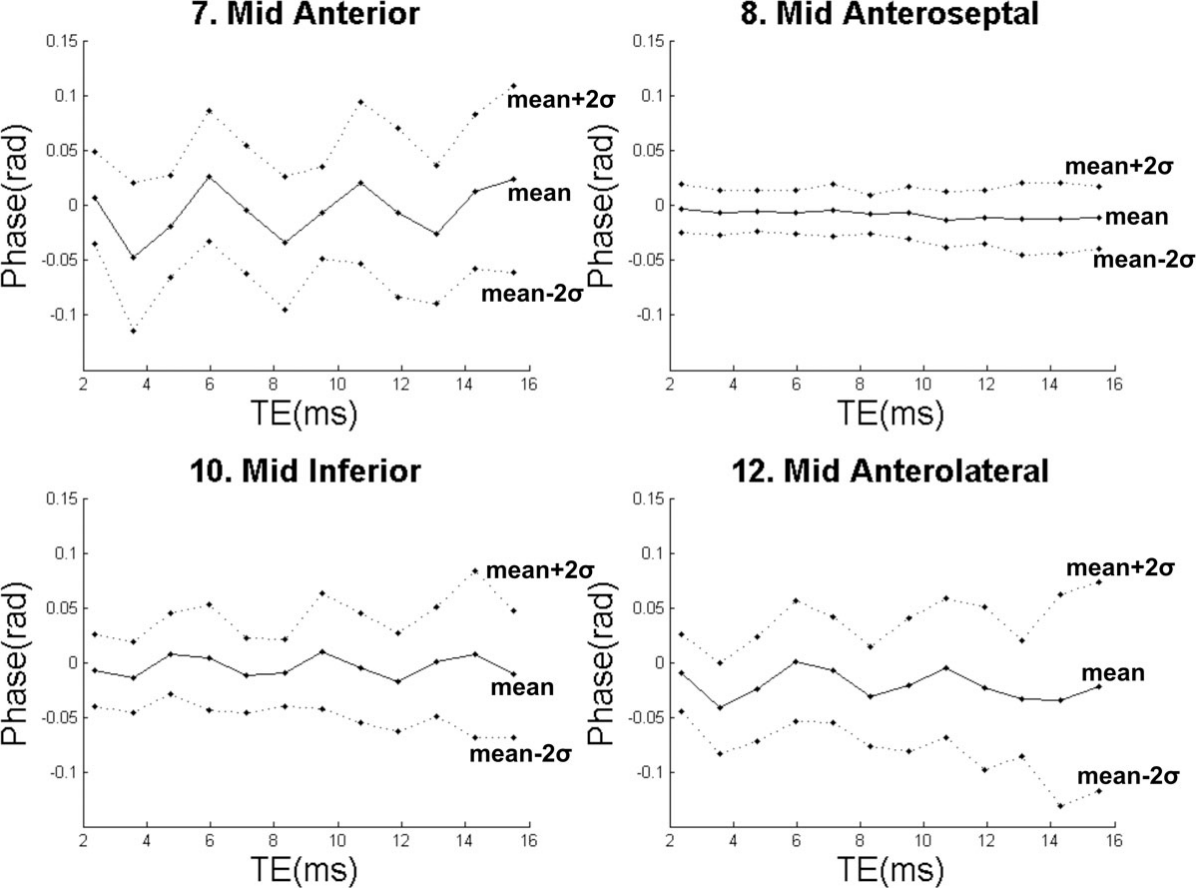
Normal phase values in 4 of 16 measured anatomical segments. Phase variance was less in all septal segments. There was a consistent cyclic variation due to fat in anterior, inferior and lateral segments.

**Figure 2:**
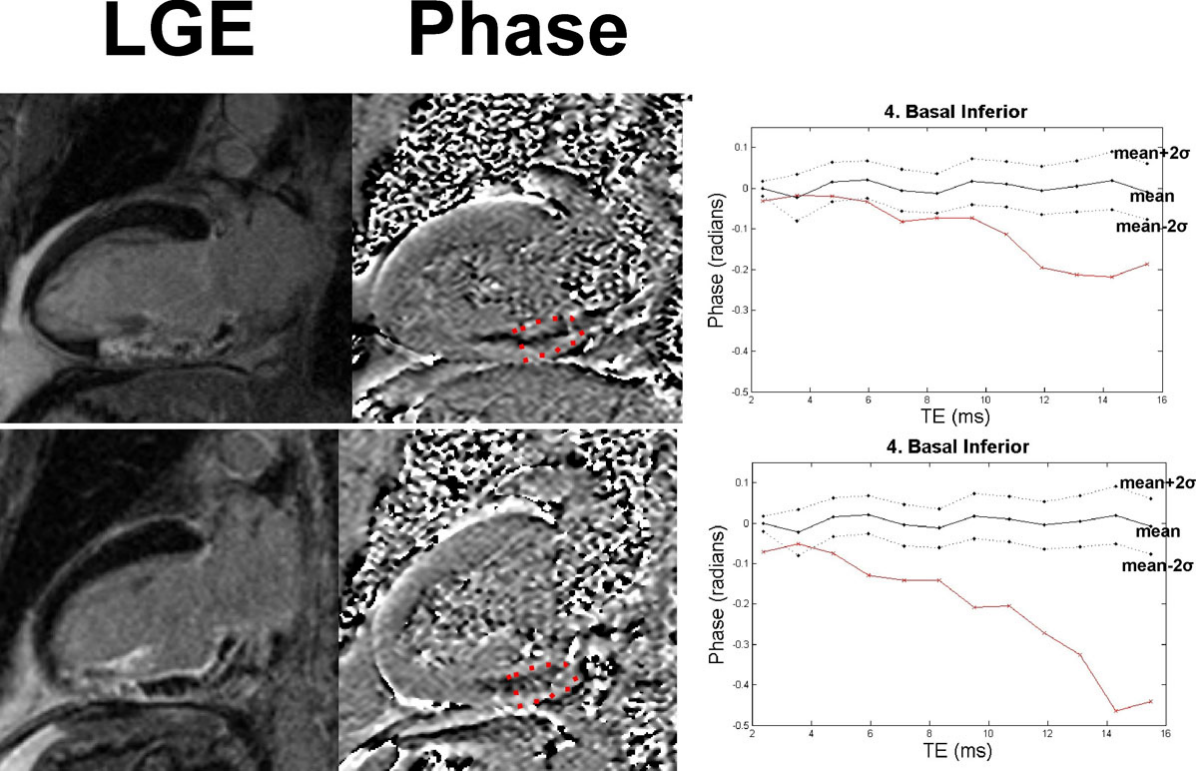
Examples from two acute MI patients with inferior infarcts, microvascular obstruction and myocardial hemorrhage. A marked negative phase is well depicted in the phase images and easily identified outside of the normal range using ROI analysis.

Filtered phase in the control group was small, on average across echo times and patients being zero in all segments with variance significantly increasing with echo time (p<0.001). There was a difference between anatomical segments (p<0.001), with less variation in septal segments compared to a cyclic variation due to myocardial fat in other segments (Figure 1). There were nine patients with transmural infarcts, 6 with MVO and 4 with hemorrhagic infarction detected by phase outside of the normal range (Figure 2) for more than four consecutive phase images. Phase in hemorrhagic infarcts decreased with echo delay time, making image contrast greater.

## Conclusions

High pass filtered myocardial phase is small and normally varies by anatomical myocardial segment and echo delay time. Myocardial hemorrhage causes a significant phase decrease beyond these normal variations. High-pass filtered phase imaging represents a quantitative, high quality method for the detection of myocardial hemorrhage without the need for the presence or high quality visualization of myocardial edema.

## Funding

American Heart Association Scientist Development Grant, 0635029N.

